# Analysis of the antibacterial effect of an *Edwardsiella tarda* LuxS inhibitor

**DOI:** 10.1186/s40064-016-1733-4

**Published:** 2016-01-28

**Authors:** Boguang Sun, Min Zhang

**Affiliations:** Key Laboratory of Experimental Marine Biology, Institute of Oceanology, Chinese Academy of Sciences, 7 Nanhai Road, Qingdao, 266071 China; College of Marine Science and Engineering, Qingdao Agricultural University, 700 Changcheng Road, Qingdao, 266109 China

**Keywords:** AI-2, *Edwardsiella tarda*, LuxS, Quorum sensing, Peptide

## Abstract

LuxS/AI-2 quorum sensing is involved in the virulence of many bacterial pathogens, including the fish pathogen *Edwardsiella tarda*. In a previous study, we identified a small peptide, 5906, which inhibits *E. tarda* LuxS activity by binding specifically to LuxS in a manner that probably prevents the formation of functional LuxS homodimer. In the present study, using Japanese flounder as the experimental animal, we analyzed the antibacterial effect of 5906 produced by DH5α/p5906 (an *Escherichia coli* strain that produces 5906) and pID5906 (a mammalian plasmid that functional in flounder constitutively expresses 5906) against different bacterial fish pathogens. We found that fish administered with both DH5α/p5906 and pID5906 exhibited reduced bacterial recovery following *E. tarda* challenge. We also examined the effect of 5906 on the infection caused by another two fish pathogen, *Aeromonas hydrophila* and *Vibrio harveyi.* The results indicated that 5906 produced by DH5α/p5906 inhibited the AI-2 activity of *A. hydrophila* and *V. harveyi*, and that fish administered with DH5α/p5906 showed enhanced resistance against challenges with both bacteria. These results suggest that 5906 or its analogues/derivatives may be exploited for the development of broad-spectrum antibacterial agents applied in the prevention and control of fish bacterial diseases.

## Background

In bacteria, quorum sensing is a process of intercellular communication through the production of and response to extracellular small signaling molecules called autoinducers (González and Keshavan 2006; Henke and Bassler 2004). Autoinducer-2 (AI-2) represents one type of autoinducers that have been discovered to date. AI-2, a furanosyl-borate-diester as it is found in *Vibrio harveyi*, is synthesized from S-adenosylhomocysteine (SAH) in two enzymatic steps involving the nucleosidase Pfs, which converts SAH to S-ribosylhomocysteine (SRH), and LuxS, which catalyzes the cleavage of the thioether linkage of SRH to produce 4,5-dihydroxy-2,3-pentanedione, the immediate precursor of AI-2 (Lewis et al. 2001; Schauder et al. 2001; De Keersmaecker et al. 2006). LuxS, the AI-2 synthase, has been identified in a wide range of Gram-negative and -positive bacteria (De Keersmaecker et al. 2006). This phenomenon, together with the discovery that the AI-2 molecule produced by one bacterial species can be sensed by bacteria of different species (Bassler et al. 1997; Surette et al. 1999), led to the proposition that AI-2 is probably a universal signaling molecule that functions in interspecies communication. The biological importance of the LuxS/AI-2 quorum sensing system has been demonstrated by numerous experimental evidences, which showed that LuxS/AI-2 is involved in physiological processes such as biofilm formation, conjugation, and sporulation (DeLisa et al. 2001; McNab et al. 2003; Barrios et al. 2006; Herzberg et al. 2006; Rickard et al. 2006; Li et al. 2007). For pathogenic bacteria, mutation of *luxS* has been found to affect bacterial virulence (Lyon et al. 2001; Ohtani et al. 2002; Joyce et al. 2004; Wang et al. 2011). Given its importance in bacterial pathogenicity, LuxS/AI-2 quorum system has been studied as a target for the development of antibacterial compounds by many research groups (Bjarnsholt and Givskov 2008; Janssens et al. 2008), and quorum sensing inhibitors with therapeutic potentials have been identified for several human bacterial pathogens, such as *Pseudomonas aeruginosa* (Hentzer et al. 2003; Wu et al. 2004; Bjarnsholt et al. 2005; Rasmussen et al. 2005; Hoffmann et al. 2007), *Staphylococcus aureus*, and *Staphylococcus epidermidis* (Mayville et al. 1999; Yang et al. 2003; Dell’Acqua et al. 2004; Kiran et al. 2008). Quorum sensing inhibitory compounds against various bacterial aquaculture pathogens have been widely studied. The research on these compounds, either natural products from marine bacteria and algae or synthetic derivatives, could be considered a major part of the effort to promote antivirulence therapy for aquaculture (Defoirdt 2014). However, quorum sensing inhibitors with peptide nature has been less reported in bacterial fish pathogens.

*Edwardsiella tarda* is a Gram-negative pathogen that can infect many freshwater and marine fish species (Xu and Zhang 2014). Fish infected by *E. tarda* develop a systematic disease called edwardsiellosis, which often leads to high mortality, especially under stress caused by poor husbandry and weather conditions. In an effort to elucidate the virulence mechanism of *E. tarda*, we found that the LuxS/AI-2 quorum sensing system is required for optimal bacterial pathogenicity (Zhang et al. 2008a). Given the essentialness of LuxS to the infectivity of *E. tarda*, we, in another study (Zhang et al. 2009), investigated the possibility of mitigating *E. tarda* infection by blocking LuxS/AI-2 signal transduction pathway through the action of LuxS inhibitors. We found that two small peptides, 5411 and 5906, which bear homology with the catalytic site of *E. tarda* LuxS, can bind to and inhibit the enzymatic activity of LuxS, whereby reducing the production of AI-2 and vitiating the virulence potential of *E. tarda*.

The present work is aimed to evaluate the potential of 5906 type peptide to develop novel antibacterial agents for use in fish farming. For this purpose, we chose 5906, which seemed more effective than 5411 on restricting bacterial dissemination in vivo when produce by an *E. tarda* source (Zhang et al. 2009), to analyze its effect on bacterial infection. Our results indicated that 5906 exhibited broad-spectrum antibacterial activities, therefore 5906 or its analogues/derivatives may have the potential for developing novel antimicrobial agents applied in fish aquaculture.

## Results and discussion

### Anti-*E. tarda* effect of 5906 produced by an *E. coli* strain

**Fig. 1:**
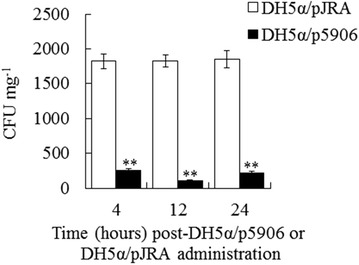
Effect of DH5α/p5906 on *E. tarda* infection. Japanese flounder were administered via intraperitoneal injection with DH5α/p5906 (*filled square*) or DH5α/pJRA (*open square*). The fish were challenged with *E. tarda* after 4, 12, and 24 h post-DH5α/p5906 or DH5α/pJRA administration and examined for bacterial recovery from liver. Data are presented as mean ± SE (N = 5). **P < 0.01

Japanese founder were administered via intraperitoneal (i.p.) injection with DH5α/p5906 which produces 5906, or the control strain DH5α/pJRA, which is DH5α harboring pJRA, the parental plasmid from which p5906 was derived. The fish were subsequently challenged with *E. tarda* TX1 at 4, 12h, and 24 h post-DH5α/p5906 or DH5α/pJRA administration and examined for bacterial recovery from the liver. The results showed that bacterial recoveries from DH5α/p5906-administered fish at all the examined time points were significantly lower than those from DH5α/pJRA-administered fish (Fig. 1).

In a previous study, we have shown that DH5α/p5906, which constitutively expresses 5906 (encoded by the plasmid p5906), can produce and secrete this peptide into the culture supernatant. Moreover, when *E. tarda* TX1 was present in the vicinity, 5906 secreted by DH5α/p5906 could, through unknown mechanisms, pass into *E. tarda* and inhibit LuxS activity, resulting in reduced production of AI-2 (Zhang et al. 2009). In the present study, we examined the possibility that, since LuxS is required for the virulence of *E. tarda*, DH5α/p5906, if administered into fish, may inhibit *E. tarda* infection by interfering with the activity of LuxS. Our results indicated that DH5α/p5906 administration significantly restricted the dissemination of TX1 in fish tissues, suggesting a potential use of heterogeneously expressed 5906 for the development of novel countermeasures against *E. tarda* infection.

### Anti-*E. tarda* effect of 5906 produced by fish

**Fig. 2:**
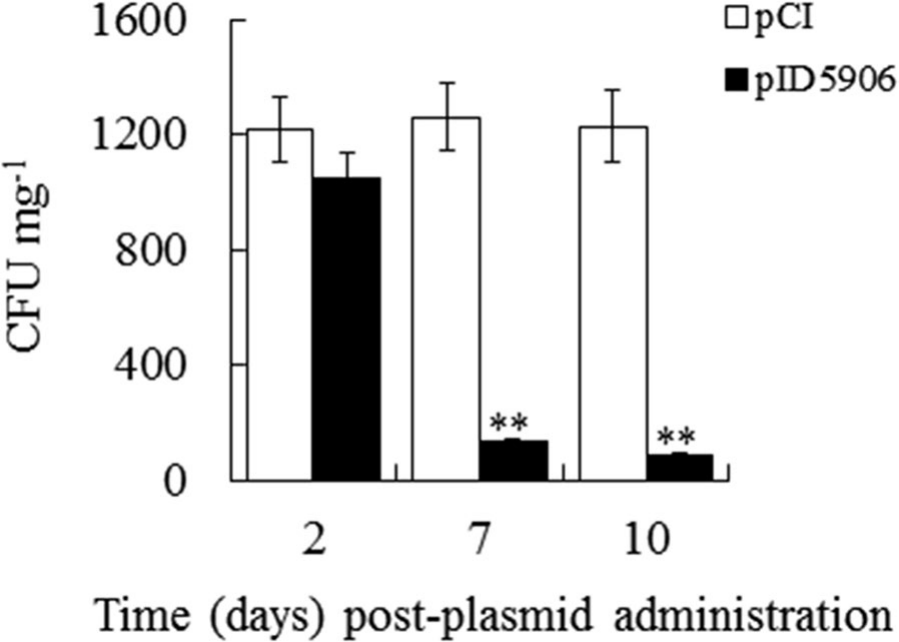
Effect of pID5906 on *E. tarda* infection. Japanese flounder were administered via muscle injection with pID5906 (*filled square*) or pCI (*open square*). The fish were challenged with *E. tarda* after 2, 7, and 10 days post-plasmid administration and examined for bacterial recovery from liver. Data are presented as mean ± SE (N = 5). **P < 0.01

To examine the effect of 5906 expressed in and produced by the animal host, the mammalian expression plasmid pID5906 was constructed, which expresses 5906 constitutively under the human cytomegalovirus immediate-early promoter. Japanese flounder were administered with pID5906 or the control plasmid parental pCI via muscle injection. The fish were challenged with *E. tarda* TX1 at 2, 7, and 10 days post-plasmid administration and examined for bacterial recovery from the liver. The results showed that bacterial recoveries from pID5906-administered fish was of comparable level to that of the control fish at 2 days post-plasmid administration but were significantly lower than those of the control fish at 7 and, especially, 10 days post-plasmid administration (Fig. 2).

**Fig. 3:**
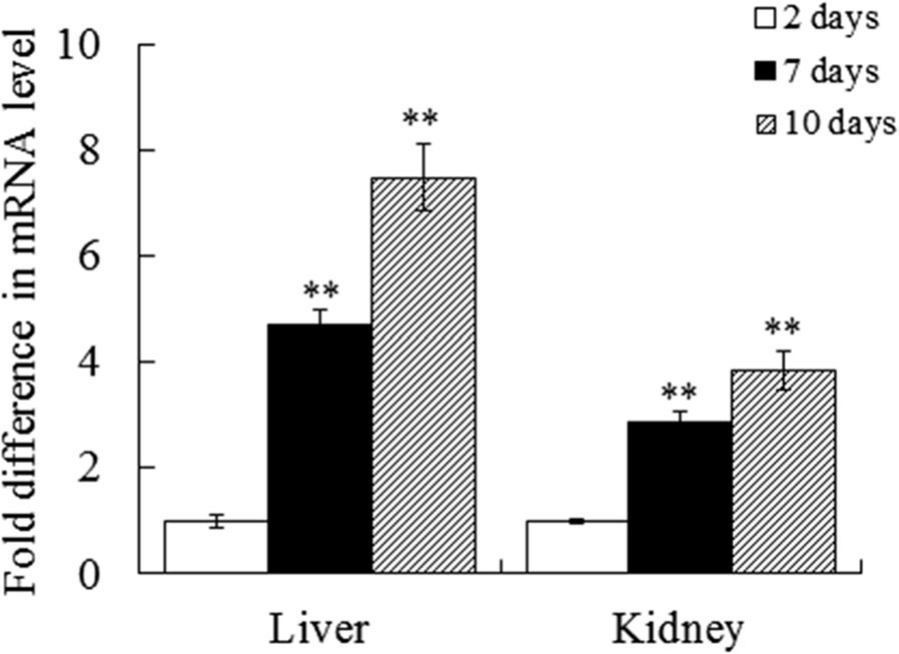
Expression of 5906 in fish organs following pID5906 administration. Japanese flounder were administered via muscle injection with pID5906 and sacrificed after 2 days (*open square*), 7 days (*filled square*), and 10 days () post-plasmid administration. 5906 expression in liver and kidney was examined by qRT-PCR. Data are presented as mean ± SE (N = 3). **P < 0.01

Consistently, qRT-PCR analysis showed that 5906 expression detected in the liver and kidney of pID5906-administered fish exhibited a time-dependent increase within the examined time frame (Fig. 3).

This observation is in agreement with the results of our previous study of *E. tarda* DNA vaccine, which showed that pCI-based plasmid could be transported to and maintained for an extended period of time in various organs following muscle injection into Japanese flounder (Jiao et al. 2009). Taken together, these results indicate that in the pID5906-administered fish, pID5906 could be transmitted to liver and kidney in a time-dependent manner and expressed therein. Upon *E. tarda* challenge, 5906 produced by the local organs probably inhibited the growth and survival of the invading pathogen by interfering with LuxS function.

### Effect of 5906 on the AI-2 activity of *A. hydrophila* and *V. harveyi*

Sequence analyses showed that *E. tarda* LuxS shares 79.9 and 74 % overall sequence identities with the LuxS of *A. hydrophila* and *V. harveyi*, respectively. The sequence of 5906, LFAGFM, which is derived from *E. tarda* LuxS, is conserved in *A. hydrophila* and *V. harveyi* LuxS, though in *V. harveyi* LuxS, the F residue at the second position of the 5906 sequence is replaced by an amino acid of kindred nature, Y. Based on these observations, we speculated that it was possible that 5906 may also be able to interact with the LuxS of *A. hydrophila* and *V. harveyi* in a fashion similar to that in which 5906 interacts with *E. tarda* LuxS. To examine this hypothesis, we determined the effect of 5906 on the AI-2 activity of *A. hydrophila* AH1 and *V. harveyi* T4, both being fish pathogens. For this purpose, AH1 and T4 were grown in LB medium supplemented with 10 % of the supernatant from a DH5α/p5906 culture, which contained secreted 5906, or from a DH5α/pJRA (control) culture. The supernatant of the above cultures was harvested at OD_600_ = 0.9 and subjected to AI-2 assay. The results showed that the AI-2 activities of AH1 and T4 grown in the presence of the culture supernatant of DH5α/p5906 were, respectively, 1.7-fold and −1.6-fold lower than those grown in the presence of the supernatant of DH5α/pJRA (results not shown). These observations suggest that 5906 in the culture supernatant of DH5α/p5906 could pass into AH1 and T4 and inhibit the production of AI-2. Our previous work has revealed that 5906 can bind specifically to LuxS of *E. tarda* TX1 and reduce the AI-2 activity of TX1, probably through intracellular interaction with LuxS (Zhang et al. 2009). Given the high homologies among LuxS of *E. tarda*, *A. hydrophila* and *V. harveyi*, it is likely that 5906 could bind and enzymatically inhibit LuxS of these bacteria via a same mechanism, which requires further investigations. Taken together, 5906 may serve as a universal quorum sensing inhibitor against different bacteria utilizing AI-2 for communication.

### Effect of 5906 on the infection of *A. hydrophila* and *V. harveyi*

**Fig. 4:**
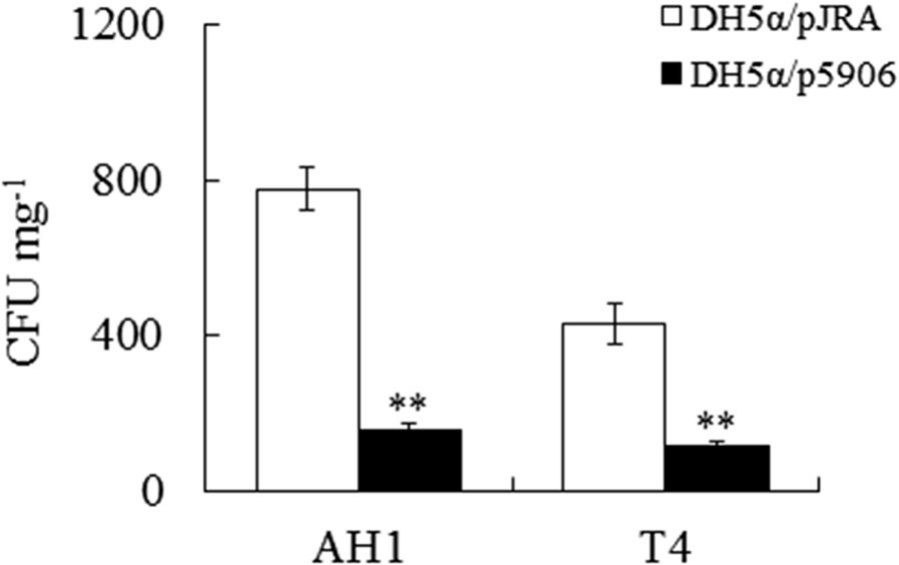
Effect of DH5α/p5906 on *A. hydrophila* and *V. harveyi* infection. Japanese flounder were administered via intraperitoneal injection with DH5α/p5906 or DH5α/pJRA. The fish were challenged with *A. hydrophila* and *V. harveyi* after 12 h post-DH5α/p5906 (*filled square*) or DH5α/pJRA (*open square*) administration and examined for bacterial recovery from liver. Data are presented as mean ± SE (N = 5). **P < 0.01

To examine whether the above observed AI-2 reduction caused by 5906 would have any effect on *A. hydrophila* and *V. harveyi* infection, DH5α/p5906 was introduced into Japanese flounder as described above, and the fish were challenged with *A. hydrophila* AH1 or *V. harveyi* T4. Subsequent bacterial recovery analysis showed that, for both *A. hydrophila* and *V. harveyi* challenges, the bacterial recoveries of DH5α/p5906-administered fish were significantly lower than those of the control fish (Fig. 4).

Compared to the effect of DH5α/p5906 on *E. tarda* infection, the effects of DH5α/p5906 on *A. hydrophila* and *V. harveyi* infections were moderate, which is likely due to the difference between *E. tarda* LuxS and *A. hydrophila* and *V. harveyi* LuxS. Since the antibacterial effect of 5906 depends on the ability of 5906 to interact with LuxS in a fashion that prevents the formation of functional LuxS, the sequence variations (compared to *E. tarda* LuxS) in *A. hydrophila* and *V. harveyi* LuxS may cause certain structural changes that compromise the recognition and/or binding of 5906.

Nevertheless, this work revealed that the small peptide inhibitor of *E. tarda* LuxS 5906 produced in *E. coli* showed effective antibacterial activity against multiple fish pathogenic bacteria. Our results imply a possibility that 5906 or its analogues/derivatives may be explored as novel antimicrobial agents applied in fish aquaculture industry. However, 5906 type inhibitors require more appropriate delivery approach to improve the applicability in fish farming. Probiotics are reported to be able to attenuate many fish pathogens (Irianto and Austin 2002), therefore becoming a promising alternative strategy to limit the indiscriminate use of antibiotics in aquaculture. A recent study reported that a probiotic *Lactobacillus sakei* strain can reduce AI-2 activity and virulence of *E. coli* 0157:H7 (Park et al. 2014). These findings enlighten us that 5906 type inhibitors may be delivered by genetically engineered probiotic bacteria strains. Although the side effects on the environmental and commensal microflora need to be carefully interrogated, a 5906-producing probiotic bacterium would provide enhanced antibacterial capacity, with reduced biosafety concerns.

## Conclusions

In this study, we analyzed in a Japanese flounder model the antibacterial effect of 5906, a small peptide that binds LuxS and interfering with LuxS activity. We found that both 5906 produced by a bacterial strain and 5906 produced by the fish host (via an expression plasmid) can effectively attenuate *E. tarda* infection. In addition to *E. tarda*, 5906 also inhibits the infection of *A. hydrophila* and *V. harveyi*. Since LuxS is highly conserved and present in diverse bacterial species, our results suggest the possibility that 5906, or its analogues/derivatives with improved potency, may be exploited for the development of broad-spectrum antibacterial agents against fish bacterial diseases.

## Methods

### Bacterial strains and growth conditions

*Edwardsiella tarda* strain TX1 (Zhang et al. 2008a), *Aeromonas hydrophila* AH1 (Wang et al. 2009), *V. harveyi* T4 (Zhang et al. 2008b), DH5α/pJRA (Zhang et al. 2009), and *Escherichia coli* DH5α/p5906 (Zhang et al. 2009) have been reported previously. All strains were cultured in Luria–Bertani (LB) medium at 37 °C (for *E. coli*) or 28 °C (for all others).

### Plasmid construction

pID5906 was created by inserting the coding sequence of 5906 (5′-AATTCACCACCATGCTATTCGCCGGCTTTATGTAACCC-3′) into pCI (Promega, Madison, WI, USA) between the *Eco*RI and *Sma*I sites.

### Fish

Japanese flounder (*Paralichthys olivaceus*, ~7 g) were purchased from a local fish farm and acclimatized in the laboratory for 2 weeks before experiments. Fish were maintained at ~22 °C in aerated seawater and fed daily with commercial dry pellets. Before experimental manipulation, fish were randomly sampled for the examination of bacterial recovery from blood, liver, spleen, and kidney, as described below. Fish were considered healthy only when no bacteria could be detected from any of the examined organs. Fish were anaesthetized by MS-222 (Sigma, St. Louis, MO, USA) before all experiments. The animal experiments were conducted in accordance with the “Regulations for the Administration of Affairs Concerning Experimental Animals” promulgated by the State Science and Technology Commission of Shandong Province.

### Bacterial recovery

Bacterial recovery from fish tissues was performed as described previously (Hu et al. 2009). Briefly, fish were sacrificed with an overdose of tricaine methanesulfonate (Sigma, St. Louis, MO, USA), and the tissues was taken under aseptic conditions. They were homogenized in phosphate-buffered saline (PBS) and plated on LB agar plates. Then the plates were incubated at 28 °C for 48 h. For bacterial detection after experimental infection, the homogenates were subjected to serial dilution before plating. The colonies emerged on the plates were enumerated. The genetic nature of the colonies was verified by PCR analysis using primers specific to *E. tarda*, *A. hydrophila*, and *V. harveyi*. PCR products were randomly selected and verified by DNA sequencing.

### The *E. tarda*-inhibitory effect of 5906 produced by an *E. coli* strain

DH5α/p5906, DH5α/pJRA, and TX1 were cultured in LB medium to mid-logarithmic phase and resuspended in PBS to 3 × 10^8^ CFU ml^−1^ (for DH5α/p5906 and DH5α/pJRA) or 8 × 10^6^ CFU ml^−1^ (for TX1). Japanese founder were randomly divided into two groups (15 fish group^−1^) and administered intraperitoneally with 100 μl of DH5α/p5906 and DH5α/pJRA, respectively. At 4 h, 12 h, and 24 h post-administration, five fish from each group were challenged with 100 μl of TX1, respectively. The fish were sacrificed at 48 h post-challenge and examined for bacterial recovery from liver as described above.

### The *E. tarda*-inhibitory effect of 5906 produced by fish

*Escherichia coli* DH5α harboring pID5906 or pCI were cultured in LB medium, and endotoxin-free plasmids were extracted using EndoFree plasmid Kit (Tiangen, Beijing, China). The purity of the purified plasmid DNA was analyzed spectrophotometrically by measuring absorbance at A_260/280_ and A_260/230_. The integrity of the plasmid DNA was assessed by agarose gel electrophoresis. Japanese founder were randomly divided into two groups (15 fish group^−1^) and administered via muscle injection with 12 μg of pID5906 and pCI, respectively. At 2, 7, and 10 days post administration, five fish from each group were challenged with TX1, respectively. Bacterial recovery from fish liver was determined at 48 h post-TX1 challenge.

### Quantitative real time reverse transcriptase-PCR (qRT-PCR)

Total RNA was extracted from fish tissues and used for cDNA synthesis as described previously (Zhang et al. 2008a). qRT-PCR was performed in an ABI 7300 Real-time Detection System (Applied Biosystems, Foster City, CA, USA) using the SYBR ExScript qRT-PCR Kit (Takara, Dalian, China) as described previously (Zhang et al. 2008a). Each assay was performed in triplicate with 16S rRNA as control. All data are given in terms of relative mRNA expressed as means plus or minus standard errors of the means (SE).

### AI-2 assay

AI-2 assay was performed as described previously (Zhang et al. 2008a). Briefly, bacterial strains to be tested were grown in LB broth to OD_600_ = 0.9. The cell-free supernatants were obtained by centrifugation and subsequent filtration through a 0.22 μm filter (Millipore). Bioluminescence induction was measured as follows: the overnight culture of *V. harveyi* BB170, an AI-2 sensor strain, was diluted 1:5000 in fresh AB medium (Surette et al. 1998) supplemented with the supernatant (10 %) of the strain to be tested or with the growth medium (control). Growth of BB170 was continued and light production was measured with a GloMax luminometer (Promega).

### Effect of 5906 on the infection of *A. hydrophila* and *V. harveyi*

Analysis of the effect of 5906 on the infection of *A. hydrophila* and *V. harveyi* was carried out exactly as described in “The *E. tarda*-inhibitory effect of 5906 produced by an *E. coli* strain” section, except that the challenging organisms were *A. hydrophila* AH1 and *V. harveyi* T4, and the challenging time was at 12 h post-DH5α/p5906 and DH5α/pJRA administration.

### Statistical analysis

All statistical analyses were performed using SPSS 15.0 software (SPSS Inc., Chicago, IL, USA). In all cases, significance was defined as *P* < 0.05.
